# G3BP1 is a SWI/SNF-bound regulator of transcription that modulates activation of STATs

**DOI:** 10.1042/BSR20250290

**Published:** 2026-05-06

**Authors:** Natalia Papadopoulos, Niki Sarri, Johan Lennartsson, Carl-Henrik Heldin

**Affiliations:** 1 Department of Medical Biochemistry and Microbiology, Uppsala University, Sweden; 2 Department of Pharmaceutical Biosciences, Uppsala University, Sweden

**Keywords:** cell proliferation, chromatin remodeling, G3BP1, PDGFRbeta, signal transducers and activators of transcription, transcription factors

## Abstract

RAS GTPase-activating protein-binding protein 1 (G3BP1) is a component of the RAS signaling pathway and a phosphorylation-dependent RNA/DNA endoribonuclease that links signal transduction and RNA metabolism. We identified G3BP1 as a nuclear interactor of platelet-derived growth factor receptor-β, forming a complex with BAF155, a component of the SWI/SNF chromatin remodeling complex, as well as with the transcription factor STAT3. Depletion of G3BP1 in human primary fibroblasts AG1523 reduced PDGF-BB-induced activation of STAT3 while increasing mRNA levels of *FOS, MYC*, and *CCND1*, which encode proteins involved in growth stimulation. Both STAT1 and cyclin D1 mRNA and protein levels were elevated upon G3BP1 knockdown, identifying G3BP1 as a negative regulator of STAT1 and cyclin D1 expression. G3BP1 depletion did not abolish PDGF-BB-induced proliferation of human primary fibroblasts. Thus, G3BP1 interacts with the SWI/SNF chromatin remodeling complex in the nucleus and regulates cell growth pathways by modulating STAT signaling and transcription of growth-associated genes.

## Introduction

Platelet-derived growth factors (PDGFs) play prominent roles in cell growth, proliferation, and migration [1]. PDGF isoforms bind to two related tyrosine kinase receptors, i.e., PDGF receptor (PDGFR) α and β. Upon activation by PDGFs, PDGF receptors dimerize and undergo auto-phosphorylation on tyrosine residues in the intracellular domain, subsequently activating downstream signaling pathways, including RAS/RAF/MEK/ERK mitogen-activated protein kinase (MAPK), phosphatidylinositol-3-kinase (PI3K)/AKT, and signal transducers and activators of transcription (STATs) [1]. STATs are latent transcription factors located in the cytoplasm that are rapidly activated in response to signals, form homo- and heterodimers, and translocate to the nucleus. STAT1 is activated by many growth factors but is primarily important for interferon-γ-dependent signaling pathways [2]. STATs can bind directly to activated PDGFRs and are phosphorylated on tyrosine residues by the receptor tyrosine kinase itself [3] or by other non-receptor tyrosine kinases, such as SRC or JAK [4]. It was shown that PDGFRβ can induce tyrosine phosphorylation of STAT1, STAT3, and STAT5, which was accompanied by specific DNA-binding activities [5]. Activation and overexpression of STAT3 have been linked to cancer [6], which could be attributed to its ability to sustain self-renewal of pluripotent stem cells [7] and, therefore, promote stem cell properties of cancer cells and chemotherapy resistance [8]. In this context, the binding of STAT3 across the pluripotent genome was shown to be dependent on an interchangeable ATPase component of the SWI/SNF remodeling complex, BRG1 [9].

SWI/SNF is a multicomponent chromatin remodeling complex with ATPase activity, which confers both activating and repressing transcriptional effects on different target genes depending on the cellular context [10]. The complex consists of two core elements with molecular masses of 155 and 170 kDa that are referred to as Brahma-related genes (BRG1)/Brahma (BRM)-associated factors, i.e., BAF155 and BAF170, interchangeable ATPase components Brg1 and BRM, and variable auxiliary modulatory subunits. These complexes have essential roles during lineage specification and in the maintenance of stem cell pluripotency [11]. Inactivating mutations in several SWI/SNF subunits have been identified in approximately 20% of a variety of cancers, exhibiting a broad mutational pattern, similar to p53 [12].

GTPase-activating protein-binding protein 1 (G3BP1) is a RAS-GAP-associated RNA/DNA endoribonuclease and the main component of stress granules that binds RNAs and ribosomal subunits during assembly of stress granules upon oxidative stress. In Drosophila, G3BP1 was shown to regulate mRNA stability and translation [13]. Inactivation of the G3BP1 gene in mice led to embryonic lethality and growth retardation, the phenotype manifesting itself in massive apoptotic cell death in the central nervous system and altered expression of essential growth factors, such as IGF-II and growth arrest-specific mRNAs, such as *GAS5* [14]. G3BP1 is overexpressed in human tumors [15] and various cancer cell lines and possesses anti-viral activities [16].

Mechanistically, G3BP1 was shown to stabilize *β-catenin* mRNA in the WNT pathway [17], to stabilize *TAU* mRNA in neuronal cells [18], and to suppress mRNA expression of peripheral myelin protein PMP22, thus regulating proliferation of breast cancer cells [19]. Moreover, G3BP1 promotes activation of NF-kB and STAT3 pathways during radiation-induced senescence of primary human lung fibroblasts and is required for the establishment of a senescence-associated secretory phenotype in senescent cells [20]. Conversely, G3BP1 was found to localize at lysosomes and suppress activation of the metabolic master regulator mechanistic target of rapamycin complex 1 by amino acids and insulin [21]. Thus, it appears that G3BP1 is positioned at the junction of several cellular functions, relating to growth regulation, neurological disease, viral infection, and cancer progression [22].

G3BP1 localizes to both the cytoplasm and nucleus, while its phosphorylation at Ser149 is important for its nuclear trafficking [23]. Despite the fact that G3BP1 was isolated as a DNA and RNA/DNA unwinding enzyme with ATP- and Mg^2+^-dependent activity, being analogous to the heterogeneous nuclear ribonucleoproteins such as nucleolin [24], the nuclear function of G3BP1 has not been elucidated. In the present study, we report that G3BP1 interacts with PDGFRβ and with the SWI/SNF chromatin remodeling complex, acting as a negative regulator of expression of *FOS, MYC, cyclin D1*, and *STAT1* mRNA. However, it promotes PDGF-BB-induced activation of STAT3, thus providing differential regulation of proliferative responses in the cell. The identified functional interactions of G3BP1 provide important insights into its role in the regulation of transcription and cellular response to growth factors.

## Results

### G3BP1 interacts with PDGFRβ and the SWI/SNF chromatin remodeling complex in the nuclear fraction

**Figure 1:**
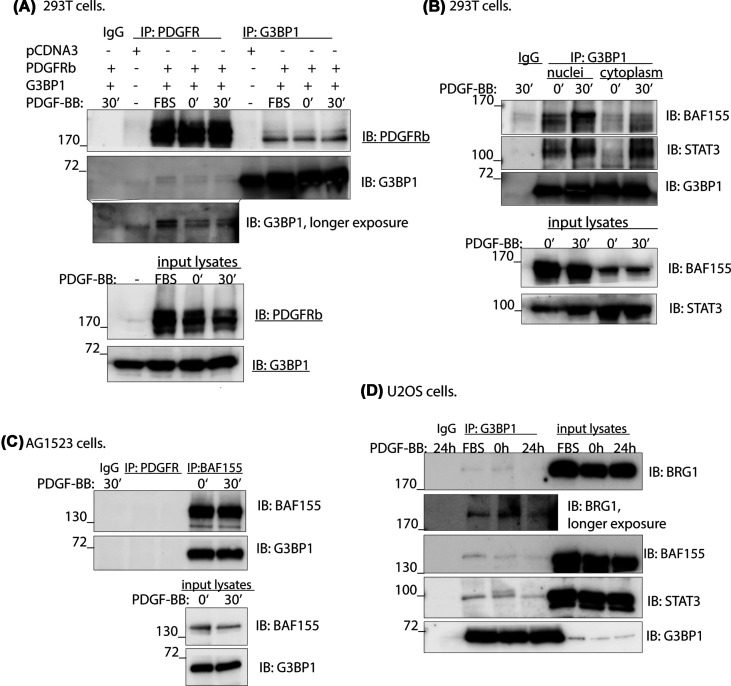
G3BP1 interacts with PDGFRβ, the transcription factor STAT3, and the SWI/SNF chromatin remodeling complex (**A**) PDGFRβ or G3BP1 was immunoprecipitated from total cell extracts of 293T cells transfected with PDGFRβ and HA-G3BP1 plasmids or with pCDNA as a negative control. Cells were serum-starved and stimulated with PDGF-BB, as indicated. Fetal bovine serum (FBS) indicates cells growing in medium supplemented with 10% FBS. (**B**) G3BP1 was immunoprecipitated from nuclear or cytoplasmic extracts of 293T cells transfected with PDGFRβ and G3BP1 plasmids and subjected to immunoblotting for BAF155, STAT3, and G3BP1. (**C**) Nuclear extracts of AG1523 fibroblasts were subjected to immunoprecipitation of endogenous PDGFRβ, BAF155, and BAF170, followed by immunoblotting for BAF155 and G3BP1. (**D**) G3BP1 was immunoprecipitated from nuclear extracts of osteosarcoma cells, followed by immunoblotting for BRG1, BAF155, STAT3, and G3BP1. The experiments represent three biological replicates for panels (A–C) and validation in the U2OS cell line (D).

G3BP1 and G3BP2 proteins were identified among nuclear interactors of PDGFRβ in a mass spectrometry screen performed on both PDGF-BB-stimulated and unstimulated cells (Table 1 and Supplementary Files S1 and S2). In this screen several members of the SWI/SNF chromatin remodeling complex were also detected, consistent with our previous findings that nuclear translocation of PDGFRβ influences the composition of the SWI/SNF chromatin remodeling complex [25]. The interaction between G3BP1 and PDGFRβ was validated in a co-immunoprecipitation experiments in 293T cells overexpressing both proteins (Figure 1A). However, the interaction between endogenous proteins in fibroblasts was not consistently detected, suggesting that it may be transient, of low affinity, or context-dependent.

**Table 1:** Selected hits from the mass spectrometry screen for nuclear interactors of PDGFRβ in unstimulated nuclear extracts

Accession	Description	Score	Coverage	# Prot	# Unique peptides	# Peptides	MW [kDa]	Calc. pI
Q13283	Ras GTPase-activating protein-binding protein 1 OS=Homo sapiens GN=G3BP1 PE=1 SV=1 - [G3BP1_HUMAN]	143.15	70.39	1	19	19	52.1	5.52
O14497	AT-rich interactive domain-containing protein 1A OS=Homo sapiens GN=ARID1A PE=1 SV= 3 - [ARI1A_HUMAN]	63.78	14.44	1	20	20	241.9	6.70
Q9UN86	Ras GTPase-activating protein-binding protein 2 OS=Homo sapiens GN=G3BP2 PE =1 SV=2 - [G3BP2_HUMAN]	47.13	34.44	1	12	12	54.1	5.55
Q969G3	SWI/SNF-related matrix-associated actin-dependent regulator of chromatin subfamily E member 1 OS=Homo sapiens GN=SMARCE1 PE=1 SV=2 - [SMCE1_HUMAN]	14.51	44.53	3	9	9	46.6	4.88
Q8TAQ2	SWI/SNF complex subunit SMARCC2 OS=Homo sapiens GN=SMARCC2 PE=1 SV=1 - [SMRC2_HUMAN]	10.96	7.91	2	11	11	132.8	5.69

Original files with full list of interactors are submitted as Supplementary File S1 (unstimulated cells) and Supplementary File S2 (30 min of stimulation with PDGF-BB).

We further investigated interactions of G3BP1 with SWI/SNF subunits and found that G3BP1 interacted with BAF155 in both 293T cells (Figure 1B) and AG1523 (Figure 1C). Additionally, we observed that G3BP1 interacted with STAT3 both in the nucleus and in the cytoplasm (Figure 1B). We also confirmed the interaction of G3BP1 with BAF155, an interchangeable ATPase component of the SWI/SNF complex, BRG1, and with STAT3 in the osteosarcoma cancer cell line U2OS (Figure 1D).

### Depletion of G3BP1 decreases PDGF-BB-induced phosphorylation of STAT3 at Tyr705

**Figure 2:**
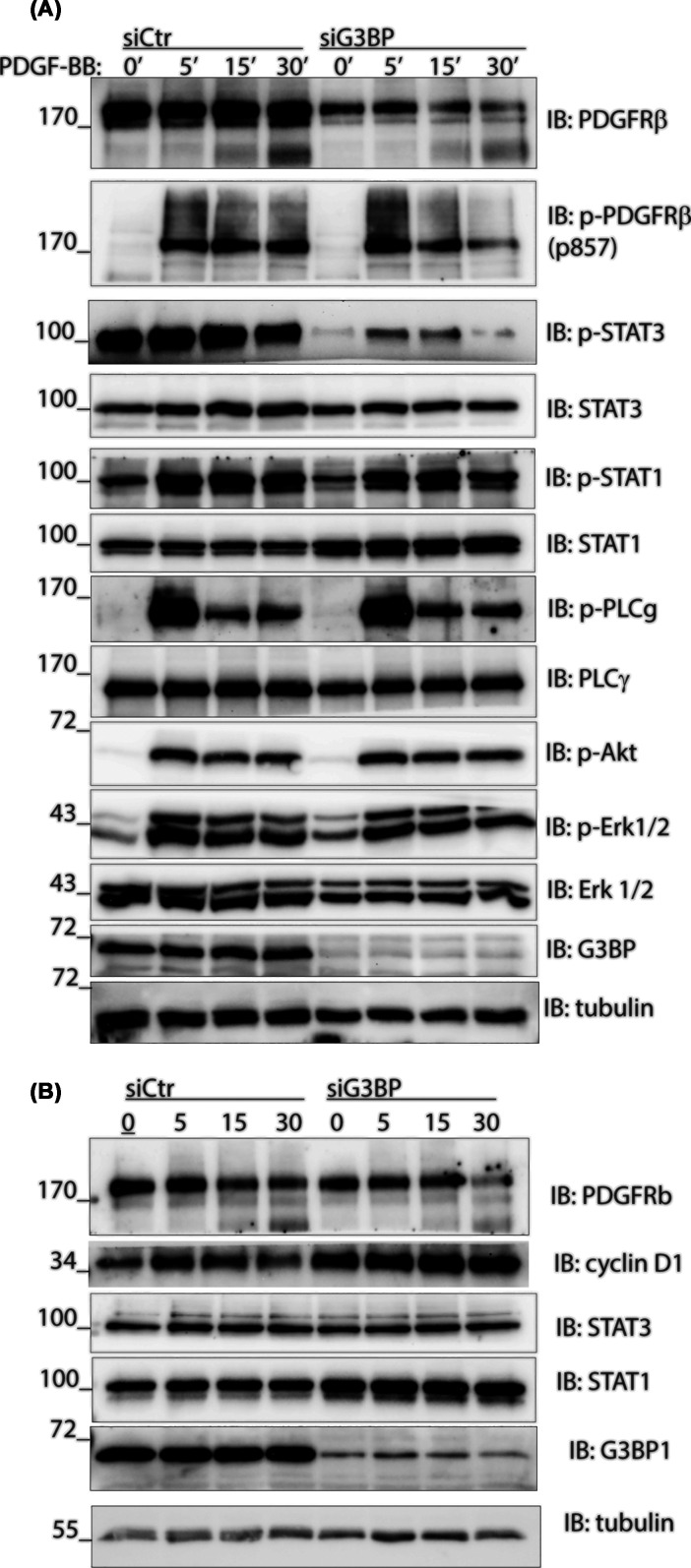
Activation of STAT3 is decreased upon G3BP1 knockdown in AG1523 fibroblasts (**A, B**) G3BP1 was transiently silenced with siRNA, or not; cells were then serum-starved and stimulated with PDGF-BB, as indicated. Activation of PDGFR, STAT1, ERK1/2, AKT, PLCγ, and STAT3 signaling pathways was determined by immunoblotting for phosphorylated and total amounts of the signaling proteins. The experiments were repeated four times.

In order to investigate the effect of G3BP1 on PDGF-BB-induced signaling, G3BP1 was depleted with siRNA in AG01523 fibroblasts, followed by stimulation with PDGF-BB for 5 to 30 min. We found that the amount of phospho-STAT3 (Tyr705) was decreased upon knockdown of G3BP1, indicating inhibition of STAT3 activation, while the total STAT3 protein level was not changed (Figure 2A). Autophosphorylation of PDGFRβ and phosphorylation of other signaling effectors, such as AKT, ERK1/2, and PLCγ, were not affected (Figure 2A). Notably, the total levels of STAT1 and cyclin D1 proteins were clearly up-regulated upon G3BP1 depletion (Figure 2B), while the amount of STAT1 phosphorylated on Tyr701 remained approximately the same, suggesting decreased stoichiometry of phosphorylation in G3BP1-depleted cells (Figure 2A).

### Lack of G3BP1 leads to an increase in mRNA levels of *STAT1, FOS*, c-*MYC*, and *cyclin D1*

**Figure 3:**
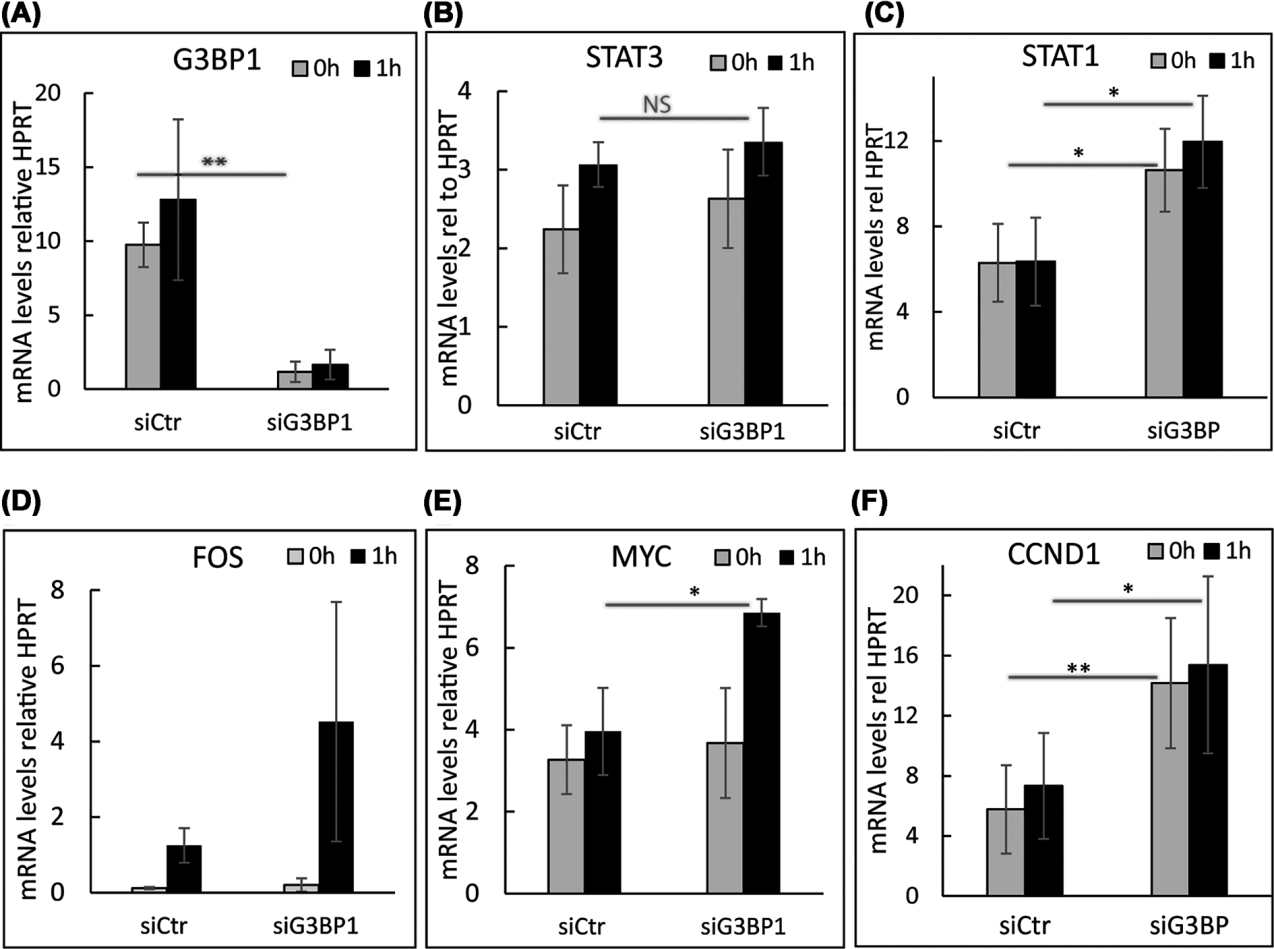
Depletion of G3BP1 up-regulates mRNA expression of *FOS, MYC, STAT1*, and *cyclin D1* (A–F) After transient silencing of G3BP1 with siRNA, or not, and serum starvation overnight, AG01523 cells were stimulated with PDGF-BB for 1 h (black bars) or left unstimulated (0 h, gray bars). mRNA expression of *G3BP1* (**A**), *STAT3* (**B**), *STAT1* (**C**), *FOS* (**D**), *MYC* (**E**), and *CCND1* (cyclin D1) (**F**) is presented relative to the expression of control gene *HPRT*. Statistical analysis was performed on four independent repeats using Student’s *t*-test. *, *P-*value <0.05; **, *P* <0.01. The experiments were repeated six times.

We proceeded to analyze the transcriptional levels of *STAT3* and *STAT1* following G3BP1 depletion (Figure 3A). Consistent with the protein expression analysis (Figure 2A,B), the *STAT3* mRNA level remained unchanged while *STAT1* expression was up-regulated (Figure 3B,C). Moreover, transient silencing of G3BP1 expression resulted in a marked increase in PDGF-BB-induced expression levels of *FOS* and *MYC*, as well as a PDGF-BB-independent increase in *cyclin D1* mRNA expression (Figure 3D–F). These findings are consistent with previous reports that G3BP1 is able to destabilize *FOS* and *MYC* mRNAs in the cytoplasm during mitogenic stimulation [23]. However, transcriptional regulation of *STAT1* and *cyclin D1* by G3BP1 has not been reported.

### Depletion of G3BP1 does not abolish PDGF-BB-induced cell proliferation

**Figure 4:**
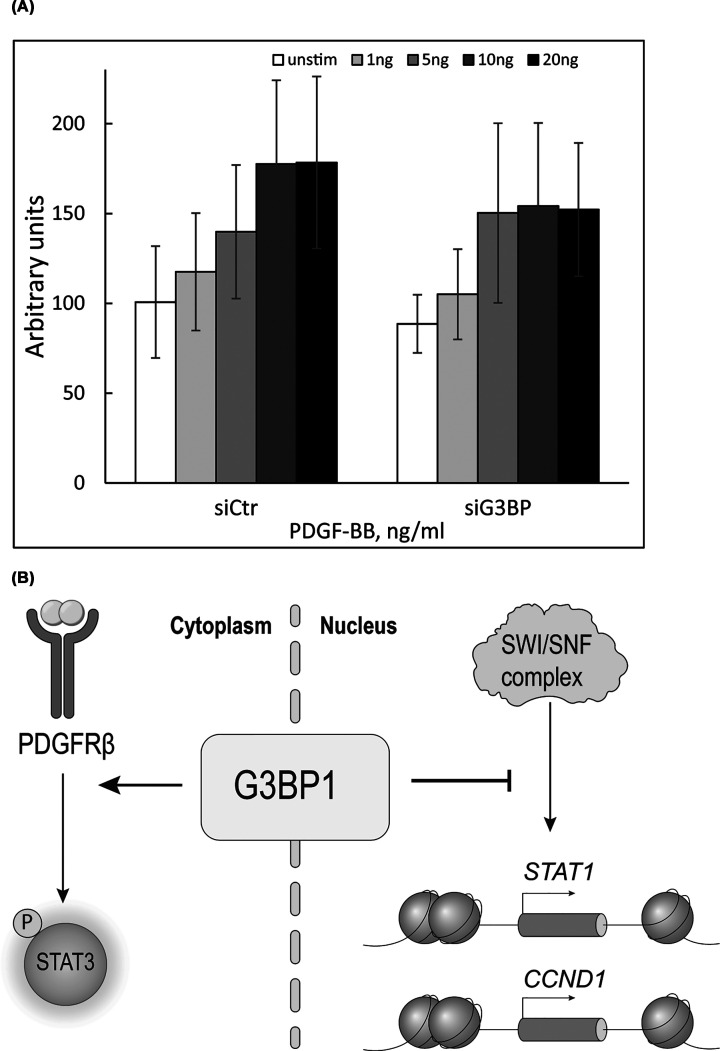
Depletion of G3BP1 does not abolish the proliferation response to PDGF-BB stimulation (**A**) G3BP1 was knocked down by siRNA, or not, in AG01523 fibroblasts. Cells were then grown in medium containing 1% FBS and increasing concentrations of PDGF-BB. The amount of DNA and, therefore, the proliferation rate was determined by measuring absorption of fluorescently labeled DNA-intercalating dye using the CyQuant assay. The experiments were repeated three times. (**B**) Schematic illustration of the findings of the study. G3BP1 acts as a co-activator of PDGF-BB-induced activation of STAT3 and as a co-repressor of the transcription of cyclin D1 and STAT1 due to its association with the SWI/SNF chromatin remodeling complex.

Since G3BP1 is overexpressed in several cancer types, we next examined the effect of G3BP1 depletion on the proliferation of primary AG01523 fibroblasts. G3BP1 knockdown resulted in a modest but non-significant reduction in cell growth compared with cells transfected with control siRNA (Figure 4A).

## Discussion

G3BP proteins are best known to play roles in the regulation of mRNA metabolism through formation of stress granules, although there is a growing body of research data strongly suggesting that G3BPs are able to bind to mRNAs outside of stress granules and regulate gene expression [22]. The precise function of G3BP1 remains unclear, as it has been reported to stabilize and enhance translation of certain mRNAs [13] while promoting degradation of certain highly structured RNAs [26].

We have previously reported that full-length cell surface PDGFRβ can accumulate in the nucleus, where the receptor modulates proliferation by affecting the composition of the SWI/SNF chromatin remodeling complex [25]. In the present study, we show that G3BP1 constitutively interacts with PDGFRβ and BAF155, a core component of the SWI/SNF complex. We found that interaction between G3BP1 and BAF155 was the same or somewhat decreased in fibroblasts and osteosarcoma cells upon stimulation with PDGF-BB, which is consistent with our previous work showing that BRG1 dissociates from the SWI/SNF complex upon stimulation with PDGF-BB. However, in transfected 293T cells the interaction between G3BP1 and BAF155 was PDGF-BB inducible, suggesting dynamic changes in the composition of the complex, which may be cell-type-dependent. We also show that BAF155 of the SWI/SNF complex interacts with STAT3, indicating that STAT3 is recruited to the SWI/SNF complex since BAF155 serves as its core subunit. This is consistent with the recent findings that STAT3 interacts with SWI/SNF in cancer stem cells [8], while the BRG1 component of SWI/SNF is required to establish chromatin accessibility at STAT3 binding targets in pluripotent embryonic stem cells [9].

G3BP1 has previously been suggested to serve as an integration point for silencing and activating signals [14]. The interaction between the G3BP1 DNA/RNA helicase and the SWI/SNF chromatin remodeling complex may explain the seemingly conflicting effects of G3BP1 on RNA metabolism, leading to either stabilization or down-regulation of different mRNAs. SWI/SNF can regulate both lineage-specific and proliferation-promoting gene expression depending on its subunit composition and cellular context. G3BP1 may therefore serve as an auxiliary component of the SWI/SNF complex, modulating transcriptional repression or activation of specific genes; however, the precise mechanism of this modulation remains to be investigated. Since BAF155 has been reported to be a specific component of SWI/SNF complexes in embryonic stem cells [27], this raises an interesting possibility that the functional connection of G3BP1 with SWI/SNF differs in embryonic and differentiated cells, which remains to be further investigated.

Additionally, we found that G3BP1 acts as a negative regulator of the PDGF-BB-induced expression of *FOS, MYC*, and *cyclin D1* mRNA but as a positive regulator of STAT3 activation, suggesting potentially opposing effects on cell proliferation. Our finding that G3BP1 is a negative regulator of PDGF-BB-induced *FOS* and *MYC* expression agrees with the previously reported ability of G3BP1 to participate in mitogen-activated decay of poly(A)^+^ mRNAs, specifically *MYC* [23], which would serve as a negative feedback mechanism in response to excessive growth stimulation.

G3BP1 has been proposed to be essential for cell survival, mainly due to its role in orchestrating the antiviral response [28] and its ability to assemble stress granules [29]. We did not find any connection to stress granules of either PDGFRβ or the SWI/SNF chromatin remodeling complex, suggesting that our findings are relevant to the function of G3BP1 in unstressed cells. Moreover, our results suggest that G3BP1 acts not only as a transcriptional regulator but also as a modulator of posttranslational modifications of target proteins, such as STAT3 phosphorylation on Tyr705, that is a hallmark of STAT3 activation. It is possible that G3BP1 facilitates STAT3 phosphorylation by acting as a scaffold protein, assembling signaling complexes or, alternatively, by sequestering kinases, as it has been shown for G3BP1 sequestering CSK, which is an inhibitory kinase for SRC family kinases in the immune synapse [30]. Depletion of G3BP1 did not affect activation of cell proliferation and cell survival signaling pathways, such as ERK1/2 MAPK and PI3K/AKT, consistent with previous reports [14,21], thus suggesting that G3BP1 affects different pathways in the cell primarily at the transcriptional level and through modulation of signaling by STATs.

Additionally, we show here that depletion of G3BP1 leads to up-regulation of total levels of STAT1 mRNA and protein, but not of STAT3. G3BP1 and G3BP2 were shown to regulate translation of interferon-stimulated genes [16], and STAT1 is important in INFγ-induced signaling [2]; thus, it is possible that G3BP1 acts as a constitutive transcriptional repressor of STAT1 in the absence of immunological stimulation. Interestingly, constitutive signaling by PDGFRβ caused lethal autoinflammation in mice, a phenotype that was dependent on STAT1 [31]. PDGF-inducible activation of STAT1 was not markedly altered, but, considering the elevated total protein levels, the stoichiometry of phosphorylation appeared reduced. This may suggest that G3BP1 serves as a common cofactor in the activation of both STAT3 and STAT1, or STATs in general.

In summary, we identified G3BP1 as a transcriptional repressor of *FOS, MYC, cyclin D1*, and *STAT1* and as a positive regulator of STAT3 in primary human fibroblasts. The association of G3BP1 with the SWI/SNF chromatin remodeling complex suggests that its transcriptional effects are mediated through this interaction. These findings provide new insights into the role of G3BP1 in the regulation of cell proliferation and may inform future strategies aimed at targeting G3BP1 in cancer [32].

## Materials and methods

### Reagents and antibodies

Polyclonal antibodies recognizing PDGFRβ (CTβ) were homemade, raised against a GST fusion of the C-terminal part of PDGFRβ [33]. Commercial primary antibodies raised against the extracellular part of PDGFRβ were obtained from R&D Systems (AF358). Primary antibodies against AKT (#9272S), phosphorylated (p) AKT (pSer473, D9E, #4060), p44/p42 MAPK (ERK1/2, 137F5, #4695), p-p44/p42 ERK1/2 MAPK (pThr202/pThr204, #9101), PLCγ1 (#2822), pPLCγ1 (pTyr783, #2821), STAT3 (79D7, #4904), pSTAT3 (pTyr705, D3A7, #9145), STAT1 (#9172), pSTAT1 (#9167), BAF155 (11956), BAF170 (#12760), and Brg1 (49360) were purchased from Cell Signaling Technology; antibodies against cyclin D1 (sc-20044) were from Santa Cruz Biotechnology; and antibodies against α-tubulin (B-5-1-2, #T6074) were from Sigma. Secondary antibodies for immunoblotting, HRP-conjugated goat anti-mouse IgG (#62–6520) and goat anti-rabbit IgG (#65–6120), were from Invitrogen.

### Cell culture

Human embryonic kidney cells 293T (ATCC) and U2OS osteosarcoma cells (kind gift of Bengt Westermark, Uppsala University) were cultured in DMEM Glutamax (Invitrogen), supplemented with 10% FBS (BioWest). Human foreskin fibroblasts AG01523 (Coriell Cell Repositories) were cultured in MEM (Sigma), supplemented with 10% FBS and 2 mM glutamine. Cells were maintained at 37°C in a 5% CO_2_ humidified incubator. Following starvation in medium containing 0.1% FBS for 3 h or overnight, cells were stimulated with 20 ng/ml PDGF-BB (Chiron Corp) for indicated periods of time.

### siRNA transfection

AG01523 cells were transiently transfected with 20 nM siRNA of Trilencer-27 G3BP siRNA (#SR, OriGene Technologies, U.S.A.) or 20 nM scrambled negative control siRNA (#SR30004, OriGene Technologies, U.S.A.), using SilentFect reagent (Bio-Rad), and incubated for 72 to 96 h at 37°C in a CO_2_ incubator.

### Mass spectrometry

Proteins were immunoprecipitated from nuclear extracts of fibroblasts with a PDGFRβ antibody cross-linked to protein G-Sepharose (Invitrogen) in 10 mM Tris, pH 8, 250 mM NaCl, and 1 mM EDTA buffer; washed five times in the same buffer; and eluted twice with 50 μl of 100 mM glycine, pH 2.7, and then neutralized to pH 7.5 with Tris. Proteins were reduced, alkylated, and on-filter digested by trypsin using 3 kDa centrifugal filters (Millipore, Ireland) according to a standard operating procedure, followed by analysis by LC-Orbitrap MS/MS (Thermo Finnigan) at the MS Facility, Uppsala University.

### Co-immunoprecipitation

Proteins were extracted with immunoprecipitation buffer (50 mM Tris, pH 7.4, 300 mM NaCl, and 1% NP-40), supplemented with 1 mM Halt^™^ protease and phosphatase inhibitor cocktail (Thermo Scientific). Crude nuclear fractions were prepared by lysing cells in isotonic buffer, collecting nuclei by centrifugation at 1000×***g*** and lysing in nuclear extraction buffer (50 mM Tris, pH 7.5, 500 mM NaCl, 0.5% Triton X-100, and 1 mM EDTA), which was subsequently diluted two-fold for co-immunoprecipitation. Cell debris was pelleted by centrifugation at 13,000 rpm for 15 min, and supernatants were incubated with the primary antibody overnight, followed by 1 h incubation with 50 μl of 50% protein G-Sepharose slurry (Thermo Scientific). The beads were washed five times with washing buffer (10 mM Tris-HCl, pH 7.4, 250 mM NaCl, and 0.5% Triton X-100), and immunoprecipitated proteins were eluted with washing buffer containing 1% sodium dodecyl sulfate (SDS).

### Immunoblotting

The protein samples were subjected to SDS–polyacrylamide gel electrophoresis and electro-transferred to PVDF membranes (Immobilon). The membranes were blocked in 5% bovine serum albumin in PBS and 0.1% Tween-20 and incubated at 4°C overnight with primary antibodies. After three washes in PBS and 0.1% Tween-20, the membranes were incubated with horseradish peroxidase-conjugated secondary antibodies for 1 h at room temperature. The proteins were visualized with the enhanced chemiluminescence detection system on a charge-coupled device camera (Bio-Rad) and quantified using Bio-Rad ImageLab 6.0.1 software.

### mRNA expression analysis

AG01523 cells were lysed, and RNA was prepared with the NucleoSpin RNA Plus kit (Macherey-Nagel) according to the manufacturer’s instructions. cDNA was prepared from 1 μg RNA using the High Capacity cDNA Kit (Applied Biosystems) and used for quantitative PCR with qPCRBio SY-green mix (PCRBio) on a CFX Opus 96 (Bio-Rad). Primer sequences are available upon request. The expression levels were calculated as the difference between the cycle threshold value for the control gene and the cycle threshold value of the test gene, taken to the power of two.

### Proliferation assay

AG1523 cells were seeded at 8000 cells per well in a 24-well plate. Cells were grown in DMEM medium containing 1% FBS for 3 additional days, stimulated or not with increasing concentrations of PDGF-BB, and then washed in PBS and frozen at −70°C. The CyQUANT Cell Proliferation assay (Thermo Scientific) was used according to the manufacturer’s instructions. The amount of DNA-intercalating fluorescent dye was measured in arbitrary units at 480 nm using an Enspire spectrophotometer (Perkin Elmer). Statistical analysis was performed on three independent repeats using Student’s *t*-test.

### Statistical analysis

All experiments were repeated three to six times, the number of biological repeats is stated in the figure legends. Statistical analysis was performed on independent biological repeats using Student’s *t*-test.

## Supplementary Material

Supplementary Files S1-S2

## Data Availability

Research data are available upon request.

## Open Access

Open access for this article was enabled by the participation of Uppsala University in an all-inclusive Read & Publish agreement with Portland Press and the Biochemical Society under an agreement with BIBSAM.

## Abbreviations

FBS: fetal bovine serum
G3BP1: GTPase-activating protein-binding protein 1
MAPK: mitogen-activated protein kinase
PDGFs: platelet-derived growth factors
PDGFR: PDGF receptor
PI3K: phosphatidylinositol-3-kinase
SDS: sodium dodecyl sulfate
STATs: signal transducers and activators of transcription
